# Sequence and phylogenetic analysis of H7N3 avian influenza viruses isolated from poultry in Pakistan 1995-2004

**DOI:** 10.1186/1743-422X-7-137

**Published:** 2010-06-24

**Authors:** Muhammad A Abbas, Erica Spackman, David E Swayne, Zaheer Ahmed, Luciana Sarmento, Naila Siddique, Khalid Naeem, Abdul Hameed, Shafqat Rehmani

**Affiliations:** 1 National Reference Laboratory for Poultry Diseases, ASI, NARC, Park Road, Islamabad 45500, Pakistan; 2 Exotic and Emerging Avian Viral Diseases Research Unit, Southeast Poultry Research Laboratory, Agricultural Research Service, U.S. Department of Agriculture, 934 College Station Road, Athens, GA 30605, USA; 3 Department of Microbiology and Biotechnology, Faculty of Biological Sciences, Quaid-I-Azam University, Islamabad 45320, Pakistan; 4 Sindh Poultry Vaccine Centre (SPVC), Animal Science Complex, Korangi, Karachi 74900, Pakistan

## Abstract

**Background:**

Avian influenza virus (AIV) infections have caused heavy economic losses to the poultry industry in Pakistan as well as numerous other regions worldwide. The first introduction of H7N3 AIV to Pakistan occurred during 1995, since then H7N3, H9N2 and H5N1 AIVs have each been sporadically isolated. This report evaluates the genetic origin of the H7N3 viruses from Pakistan collected 1995-2004 and how they disseminated within the country. To accomplish this we produced whole genome sequences for 6 H7N3 viruses and data for the HA and NA genes of an additional 7 isolates. All available sequence from H7N3 AIV from Pakistan was included in the analysis.

**Results:**

Phylogenetic analysis revealed that there were two introductions of H7 into Pakistan and one N3 introduction. Only one of the H7 introductions appears to have become established in poultry in Pakistan, while the other was isolated from two separate outbreaks 6 years apart. The data also shows that reassortment has occurred between H7N3 and H9N2 viruses in the field, likely during co-infection of poultry. Also, with the exception of these few reassortant isolates, all 8 genes in the predominant H7N3 virus lineage have evolved to be phylogenetically distinct.

**Conclusions:**

Although rigorous control measures have been implemented in commercial poultry in Pakistan, AIV is sporadically transmitted to poultry and among the different poultry industry compartments (broilers, broiler breeders, table egg layers). Since there is one primary H7 lineage which persists and that has reassorted with the H9N2 AIV in poultry, it suggests that there is a reservoir with some link commercial poultry. On a general level, this offers insight into the molecular ecology of AIV in poultry where the virus has persisted despite vaccination and biosecurity. This data also illustrates the importance of sustained surveillance for AIVs in poultry.

## Background

Avian Influenza Viruses (AIV) are among the most prominent viruses affecting animal and public health. AIV infections have caused heavy economic losses to the poultry industry world-wide. Several, sporadic outbreaks of AIV of different subtypes have occurred in Pakistan since the mid-1990's [1-3] . The first highly pathogenic (HP) AIV outbreak was observed in Pakistan in December 1994 at Salgran, near the capital city of Islamabad. The disease was controlled within 4-5 months by mass vaccination with a vaccine prepared from a field isolate [4]. Then in November 1998 an outbreak that was later identified as H9N2 low pathogenicity (LP) AIV occurred in North West Frontier Province in otherwise healthy flocks that had not been vaccinated for AIV [1,3]. In 2000-2001, another outbreak was observed in Central Pakistan (Punjab), caused by H7N3 LP AIV which was controlled by ring vaccination with an aqueous-based vaccine produced with a local AIV strain followed by administration of an oil-based vaccine. During early 2003, another outbreak of H7N3 LPAIV occurred in the Southern coastal region of the country, where more than 70% of the total commercial layer flocks in Pakistan are reared. Within months this LP AIV strain mutated to the HP form, producing a sudden increase in mortality and in November 2003 the H7N3 virus had an intravenous pathogenicity index of 2.8. A LP AIV of the H9N2 subtype, was isolated from some of the same flocks infected with the H7N3 HP AIV. This outbreak spread during the next 4 weeks, and primarily affected commercial table-egg layer flocks throughout the poultry estates in that area. The outbreak was controlled by adopting strict bio-security measures, voluntary depopulation, strategic vaccination, and the implementation of a surveillance program in poultry populated areas throughout the country [4].

This study was conducted to characterize the genomes of the H7N3 type Influenza Viruses, circulating in Pakistan from 1995 through 2004, and to elucidate the pattern of AIV spread and reassortment within the country.

## Results

### H7 Hemagglutinin gene

**Figure 1:**
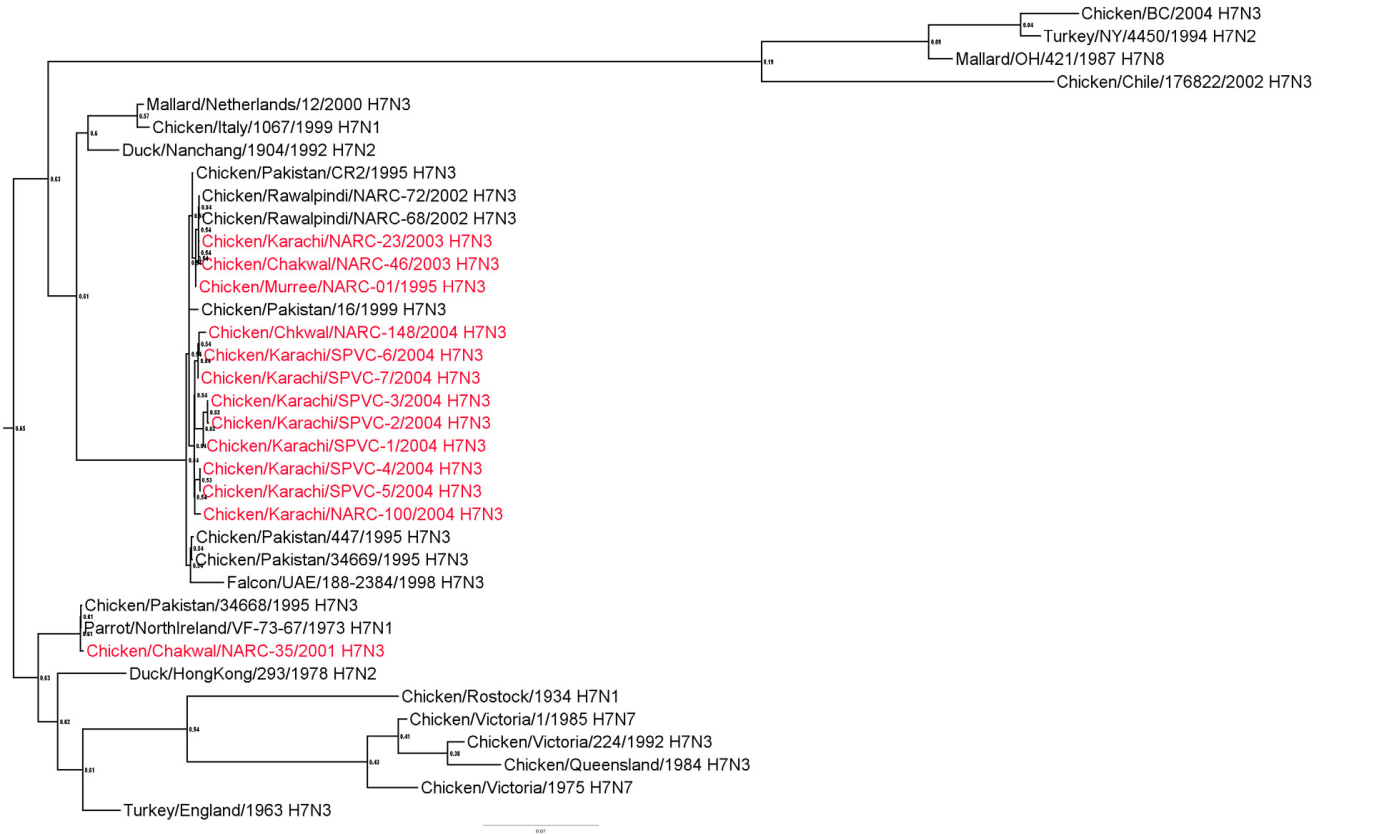
**Phylogenetic tree of the Pakistani AIV H7 HA genes and other selected AIV isolates**. The tree was constructed with merged duplicate runs of BEAST v. 1.4.8 using HKY substitution, empirical base frequency, gamma heterogeneity, codon 2 partitions, relaxed lognormal clock, Yule process tree prior with default operators with unweighted pair group mean with arithmetic average starting tree and a Markov Chain Monte Carlo length of 10^7^.

There were 2 phylogenetic groups of H7 HA genes from AIV isolates from Pakistan (abbreviations defined in Table 1). There was a major group with 18 isolates (Figure 1) with 98.3-99.9% nucleotide (nt) identity (additional file 1). A second smaller group was comprised of 2 isolates: 35/Chakwal-01 and chicken/Pakistan/34668/95 (34668/Pak-95) which had between 88.6 and 89.5% nt identity with the HA genes of the other isolates from Pakistan. These two isolates, 35/Chakwal-01 and 34668/Pak-95, were most closely related to the HA gene of A/Parrot/NorthIreland/YF73-67/1973 (H7N1) (approximately 99.7% identity). The most closely related lineages were recent viruses from poultry in China (e.g. A/Duck/Nanchang/1904/1992) and the Eurasian lineage isolated in Italy during 1997-2003 both of which had 91.0-93.0% identity with the viruses from Pakistan (Figure 1).

**Table 1:** Abbreviations used and GenBank accession numbers for H7N3 Influenza virus isolates included in this study as new sequence

Isolate	Abbreviation	HA	NA	NS	M	NP	PA	PB2	PB1
A/Chicken/Murree/NARC-01/1995	01/Murree-95	FJ577510	FJ577512	FJ577514	FJ577511	FJ577513	FJ577515	FJ577517	FJ577516
A/Chicken/Chakwal/NARC-35/2001	35/Chakwal-01	FJ577518	FJ577520	FJ577522	FJ577519	FJ577521	FJ577523	FJ577525	FJ577524
A/Chickem/Karachi/NARC-23/2003	23/Karachi-03	FJ577526	FJ577528	FJ577530	FJ577527	FJ577529	FJ577531	FJ577533	FJ577532
A/Chicken/Chakwal/NARC-46/2003	46/Chakwal-03	FJ577534	FJ577536	FJ577538	FJ577535	FJ577537	FJ577539	FJ577541	FJ577540
A/Chicken/Karachi/NARC-100/2004	100/Karachi-04	FJ577542	FJ577544	FJ577546	FJ577543	FJ577545	FJ577547	FJ577549	FJ577548
A/Chicken/Chakwal/NARC-148/2004	148/Chakwal-04	FJ577550	FJ577552	FJ577554	FJ577551	FJ577553	FJ577555	FJ577557	FJ577556
A/Chicken/Karachi/SPVC-1/2004	1/Karachi-04	FJ638319	FJ638320	-^a^	-	-	-	-	-
A/Chicken/Karachi/SPVC-2/2004	2/Karachi-04	FJ638321	FJ638322	-	-	-	-	-	-
A/Chicken/Karachi/SPVC-3/2004	3/Karachi-04	FJ638323	FJ638324	-	-	-	-	-	-
A/Chicken/Karachi/SPVC-4/2004	4/Karachi-04	FJ638325	FJ638326	-	-	-	-	-	-
A/Chicken/Karachi/SPVC-5/2004	5/Karachi-04	FJ638327	FJ638328	-	-	-	-	-	-
A/Chicken/Karachi/SPVC-6/2004	6/Karachi-04	FJ638329	FJ638330	-	-	-	-	-	-
A/Chicken/Karachi/SPVC-7/2004	7/Karachi-04	FJ638331	FJ638332	-	-	-	-	-	-

a. - No sequence data available.

Three deduced amino acid sequences for cleavage site of the HA genes from a total of 17 H7 viruses from Pakistan were observed. Thirteen of the viruses had the sequence PETPKRRK/R, two isolates (34669/Pak-95 and 447/Pak-95) had the sequence PETPKRKRK/R, and two isolates (35/Chakwal-01 and 34668/Pak-95) had the sequence PEIPKG/R. All were classified as HPAIV except 35/Chakwal-01 and 34668/Pak-95 which are LPAI viruses.

### N3 Neuraminidase gene

**Figure 2:**
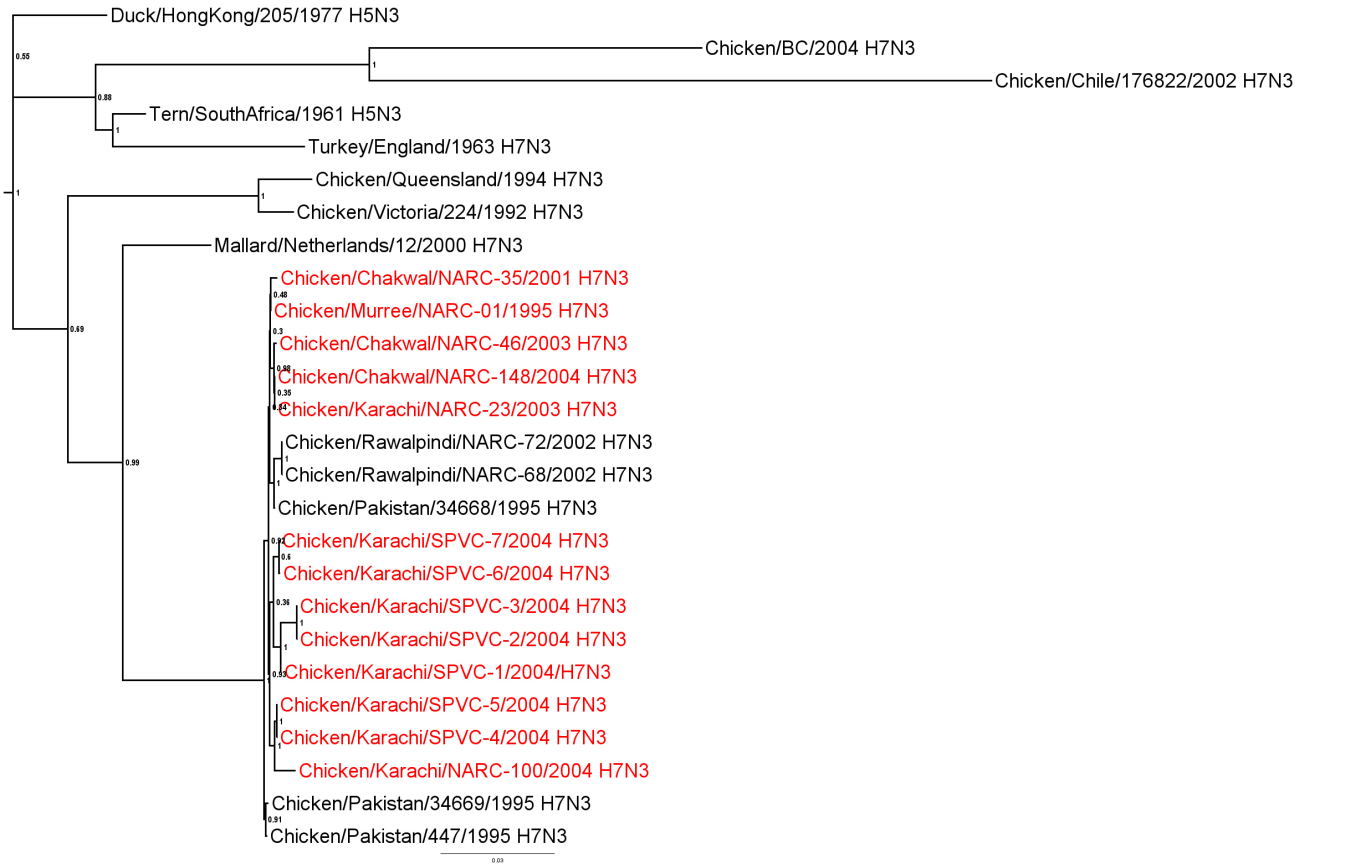
**Phylogenetic tree of the Pakistani AIV N3 NA genes and other selected AIV isolates**. The tree was constructed as described for figure 1.

All the NA genes from the Pakistani isolates were closely related to each other with 98.9-100% nt identity (additional file 2) and formed a single phylogenetic group (Figure 2). The most closely related N3 lineage with 93.9-95.0% identity were the poultry and wild bird viruses from Europe (Figure 2). All of the isolates from Pakistan had a full length NA stalk.

### Non-structural gene

**Figure 3:**
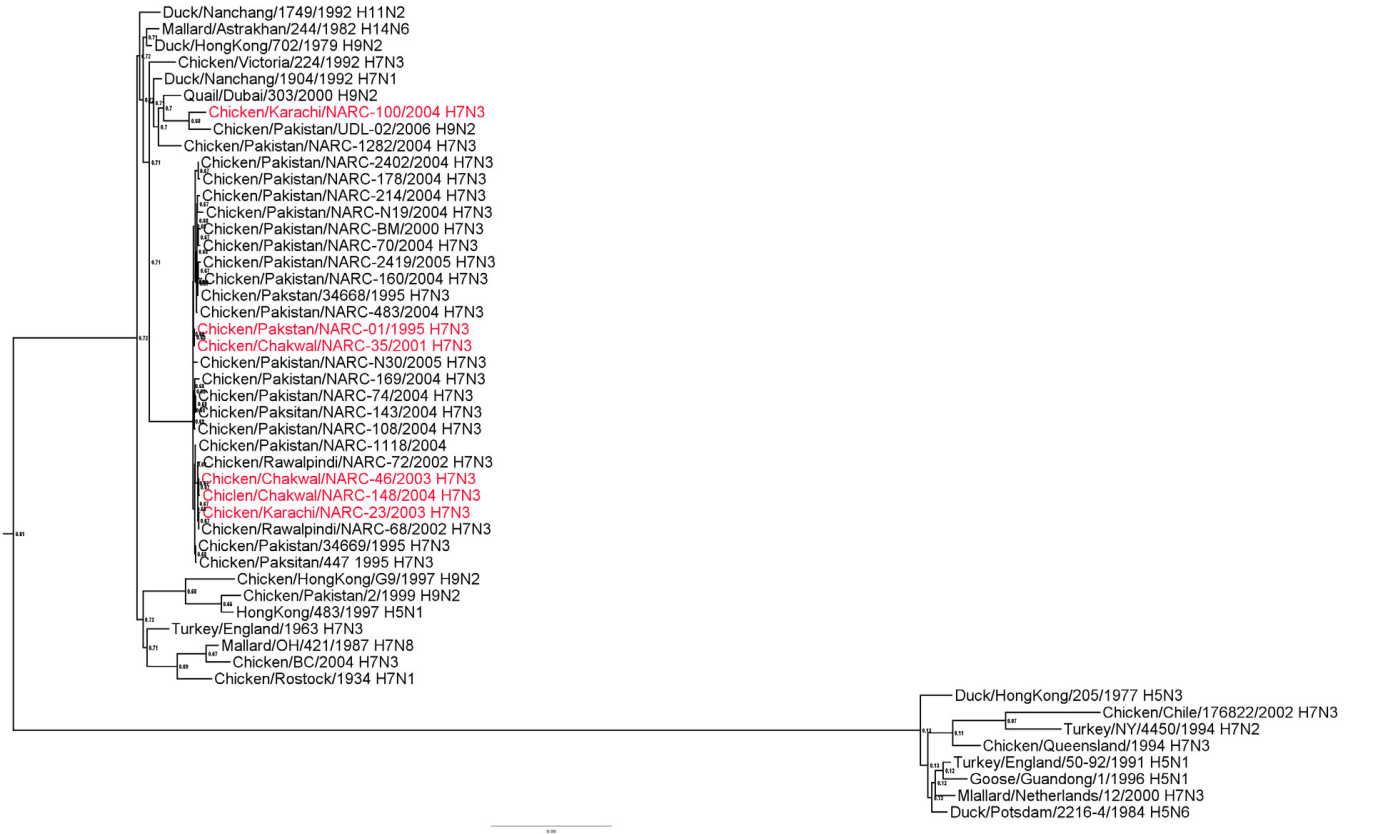
**Phylogenetic tree of the Pakistani AIV NS genes and other selected AIV isolates**. The tree was constructed as described for figure 1.

Most of the Pakistani H7N3 isolates had closely related NS genes (around 98.7-100% nt identity) (additional file 3) of which all were subtype A (Figure 3). Twenty-four encoded a truncated NS1 protein of 217 amino acids (aa). The isolates with the truncated NS1 all clustered together phylogenetically. Two isolates from the 2004 outbreak (100/Karachi-04 from Southern Pakistan, and A/Chicken/Mansehra/NARC-1282/2004 from Mansehra, Abbottabad in Northern Pakistan) encode the full-length (230 aa) NS1 gene. The NS genes from these two isolates are most closely related to the Pakistan H9N2 viruses from 2006 with around 97.0% nt identity. Another exception was 01/Murree-95, which had a 216 aa NS1 and a 120 aa NS2 protein which grouped with the other isolates with truncated NS1 proteins. The NS2 proteins of all other H7N3 isolates were a full length of 121 aa.

### Matrix gene

**Figure 4:**
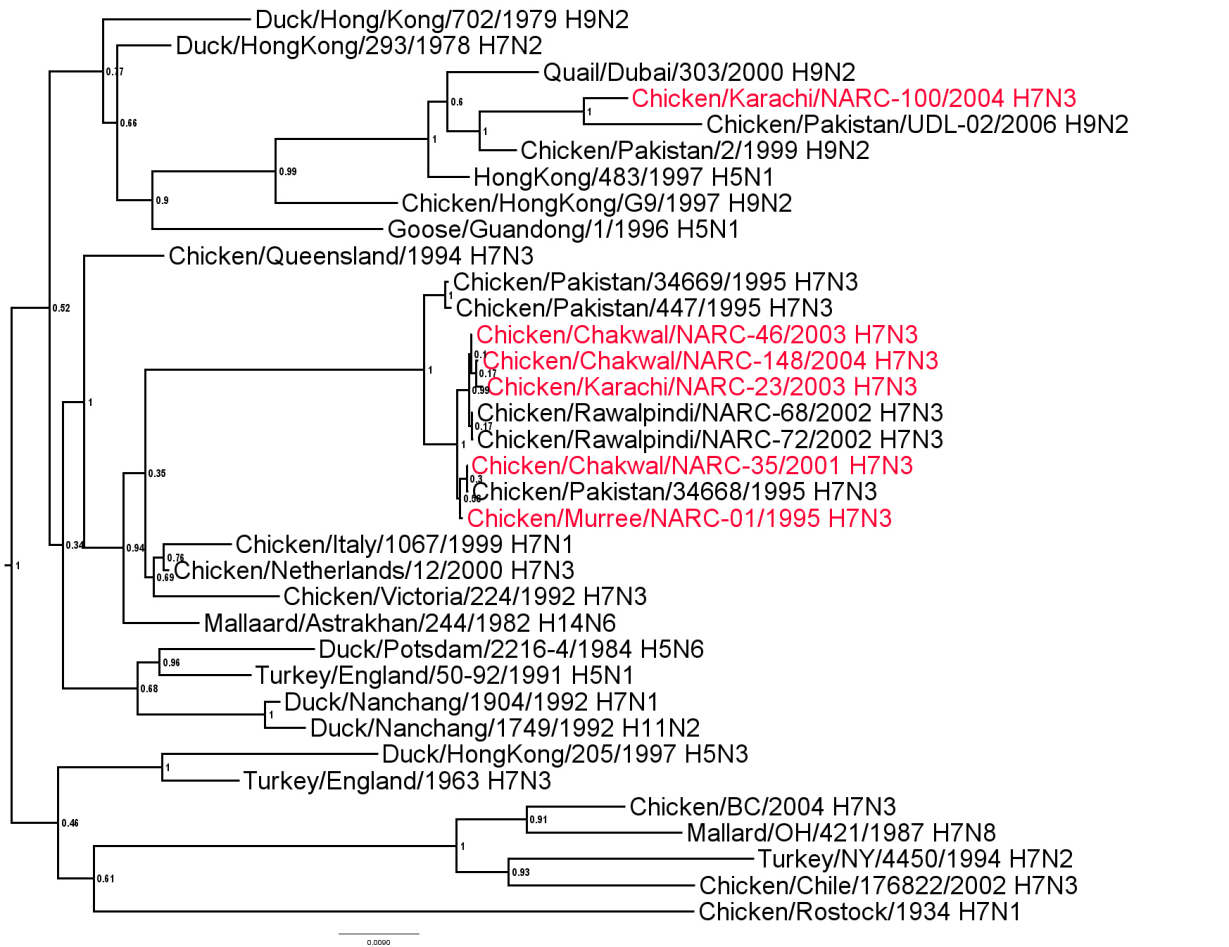
**Phylogenetic tree of the Pakistani AIV M genes and other selected AIV isolates**. The tree was constructed as described for figure 1.

The matrix genes of all the Pakistani H7N3 isolates had nt identity ranging from 99.2-100% (additional file 4) and phylogenetically assort into a single clade with the exception of 100/Karachi-04 which had about 90% identity with the other Pakistani Isolates (Figure 4). The most closely related matrix genes to 100/Karachi-04 are viruses from the Pakistan 2005-2008 H9N2 lineage which have around 97.5-98.5% nt identity with it.

### Nucleoprotein gene

**Figure 5:**
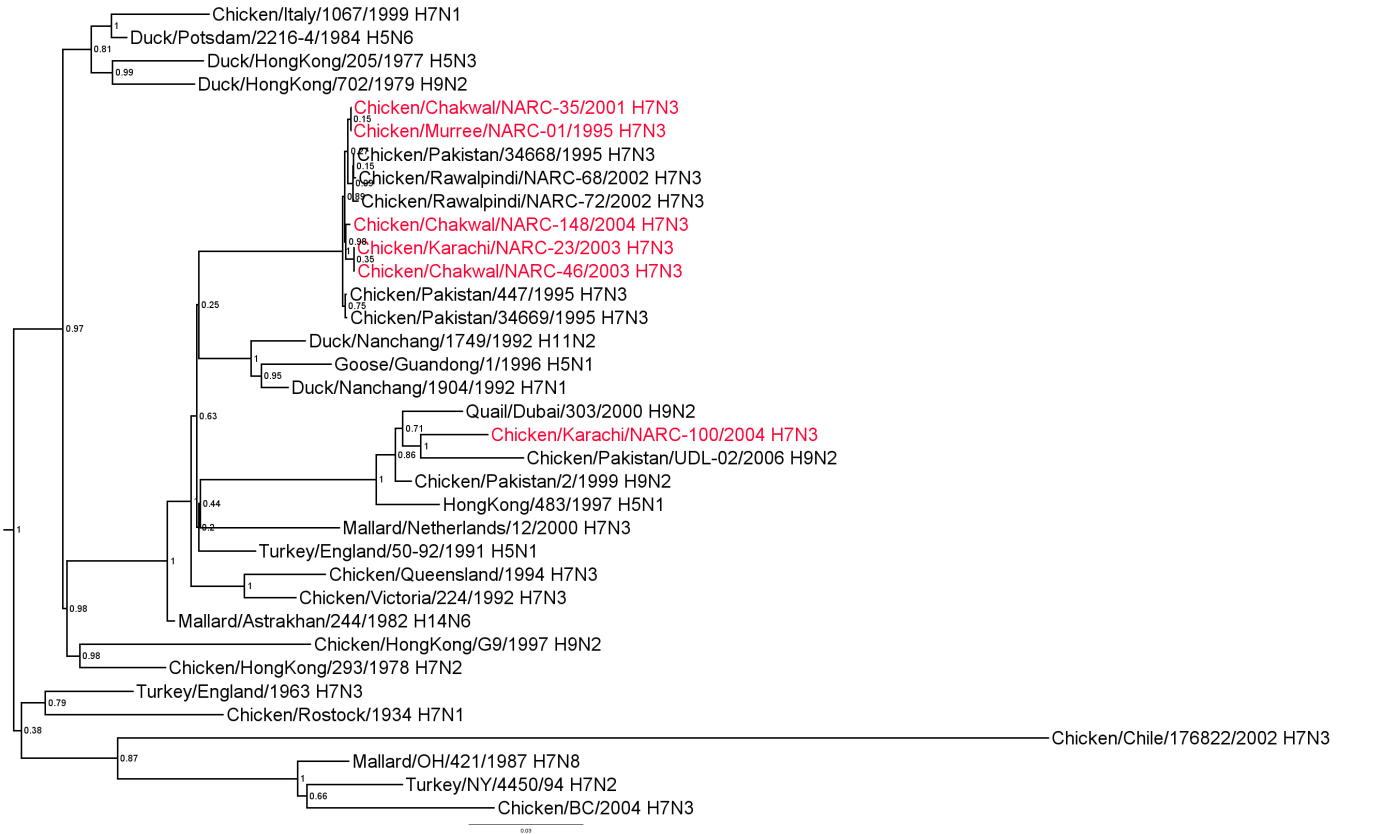
**Phylogenetic tree of the Pakistani AIV NP genes and other selected AIV isolates**. The tree was constructed as described for figure 1.

Nucleoprotein genes from the H7N3 Pakistani isolates were very closely related with nt identity above 99.7% with the exception of 100/Karachi-04 (additional file 5). The most closely related lineages to the main clade were wild bird and poultry isolates from Europe and Asia collected in the 1990's with around 95% identity (Figure 5). The isolate 100/Karachi-04 had around 91% identity with the NP genes from the other H7N3 viruses and the most closely related lineage was the 1999 and 2005-2008 H9N2 viruses from Pakistan.

### PA gene

**Figure 6:**
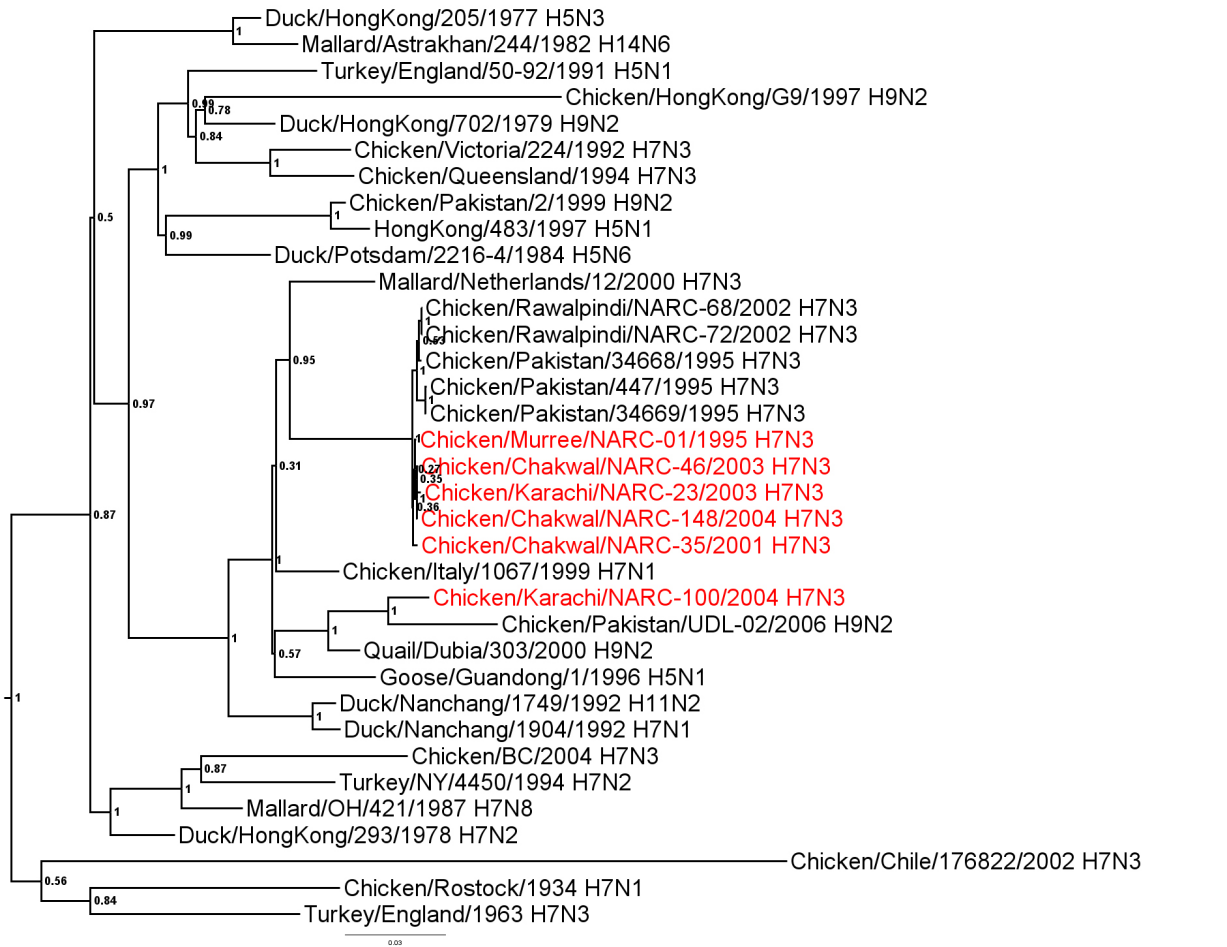
**Phylogenetic tree of the Pakistani AIV PA genes and other selected AIV isolates**. The tree was constructed as described for figure 1.

Among the PA genes from the Pakistani H7N3 isolates the nt sequence identity was above 99.5% with the exception of 100/Karachi-04 which had around 92.0% nt identity with the other H7N3 isolates (additional file 6). Phylogenetically, the NP genes of all the Pakistani H7N3 viruses except 100/Karachi-04, assorted into a single cluster (Figure 6). The most closely related lineages to all the Pakistani viruses were European duck viruses and A/Quail/Dubai/303/2000 (H9N2).

### PB1 gene

**Figure 7:**
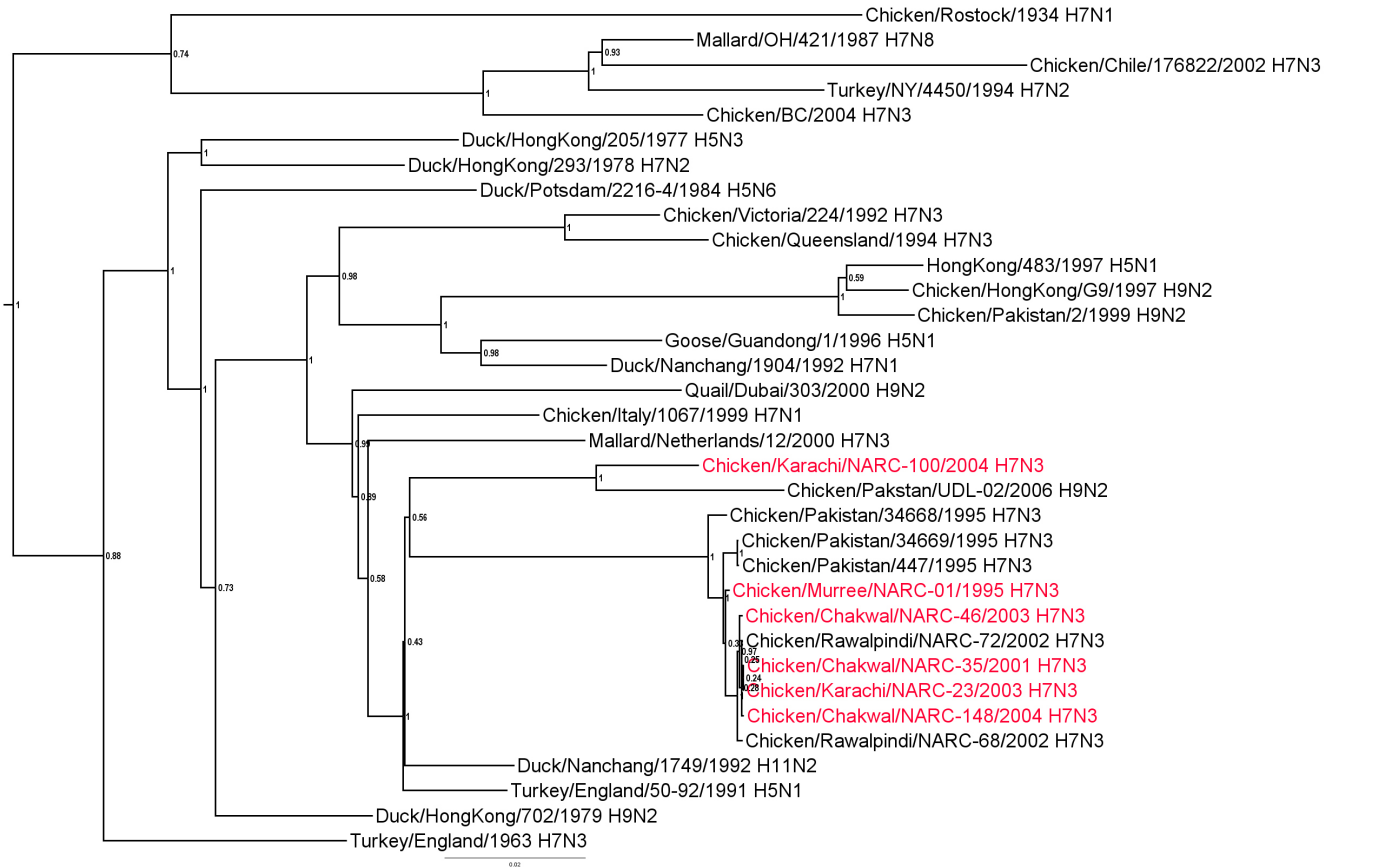
**Phylogenetic tree of the Pakistani AIV PB1 genes and other selected AIV isolates**. The tree was constructed as described for figure 1.

Nucleotide identity among the PB1 genes of Pakistani H7N3 isolates ranged from 99.6 to 100% (additional file 7), except 100/Karachi-04 which had nt identity of around of 92.7% to the other H7 isolates. Phylogenetic analysis showed that the PB1 of all the Pakistani H7N3 viruses grouped together as a distinct lineage and 100/Karachi-04 grouped with the 2005-2008 Pakistani H9N2 lineage of viruses (Figure 7).

### PB2 gene

**Figure 8:**
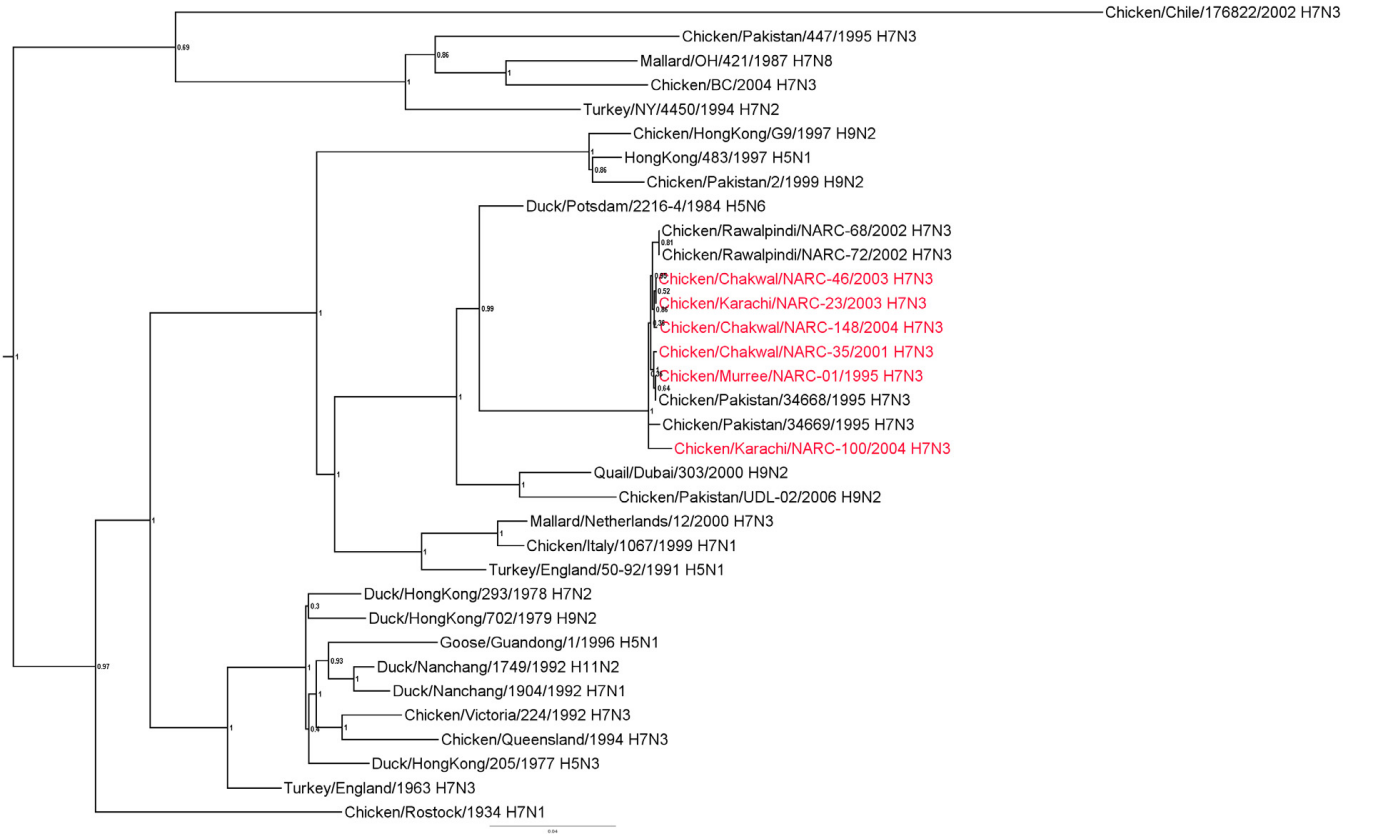
**Phylogenetic tree of the Pakistani AIV PB2 genes and other selected AIV isolates**. The tree was constructed as described for figure 1.

There was 99.1-100% nucleotide sequence identity among all the Pakistani isolates of the H7N3 subtype except chicken/Pakistan/447/1995 which had around 84% identity (additional file 8). Whereas, 92% and 90% nucleotide sequence similarity was observed with the isolates A/Quail/Dubai/303/2000 (H9N2) and A/turkey/England/50-92/1991 (H5N1), respectively which were the most related isolates from other lineages (Figure 8).

## Discussion

The first H7N3 introduction of AIV into Pakistan in 1995 was LP then after circulating for a period of 6-8 months in the poultry population a HP virus emerged [1,5]. The details of AIV in Pakistan between 1995 and 2002 are limited due to the lack of an AIV surveillance program, which was established in 2002-2003. Prior to that there were some reports of seroconversion to AIV and some sporadic isolations of LPAIV from the poultry population [6] and from domestic birds that had been exported from Pakistan [7]. Importantly, although there was no standard vaccination program, a 1995 isolate was used by various poultry producers as an inactivated vaccine until 2003. Then in 2003-2004 a more extensive outbreak of H7N3 started in Karachi, in the Southern region of Pakistan, then spread in poultry throughout the country and was controlled with a combination of vaccination and biosecurity [4].

Not surprisingly, these viruses are most closely related to AIVs from Europe, Asia and Australia, which allows for speculation about the related lineages from which the initial viruses were introduced into Pakistan based on possible common ancestors, but not the specific origin of the viruses, i.e. how and when they were introduced. All 8 genes of the H7N3 viruses from Pakistan form distinct clades (at least 4% difference in nt identity with the next most closely related isolated lineage) with these exceptions: 1) the NS, M, NP, PA and PB1 of 100/Karachi-04 which groups with the H9N2 AIVs from Pakistan, and 2) the HA genes of 34668/Pak-95 and 35/Chakwal-01 which group with A/Parrot/NorthIreland/VF-73-67/73 (H7N1); 3) the PB2 of chicken/Pakistan/447/1995 which groups with a 1985 duck virus from Canada.

This suggests that the primary lineage of H7N3 AIVs in Pakistan are the result of a single initial introduction, probably from wild birds and have circulated long enough to evolve into separate clades from other lineages of AIV, including the H9N2 lineages described previously [2,8]. In addition and typical of AIV, the virus has undergone reassortment; 100/Karachi-04 appears to have emerged by reassortment with the H9N2 poultry viruses, therefore was likely generated by concomitant infection of poultry with both lineages, rather than through a novel introduction from wild birds. This agrees with a previous report on the H9N2 AIV lineages in Pakistan by Iqbal et al. [8]. Correlating with this, during this outbreak there were several cases where viruses of both lineages were isolated from the same flock. The HA genes of 34668/Pak-95 and 35/Chakwal-01, which are most closely related to A/Parrot/NorthernIreland/VF-73-67/1973 H7N1 and other viruses from European poultry from the 1970's, are an interesting case as a possible epidemiological link is not obvious. Lastly there is the PB2 gene of chicken/Pakistan/447/1995 which is most closely related to the PB2 of duck/Alberta/228/1985, which is suggestive of a reassortment event with wild bird origin AIVs.

Also, the majority of the H7N3 viruses have some genetic features which are consistent with adaptation to poultry, an HA proteolytic cleavage site (PCS) sequence with multiple basic amino acids [2,9,10] and a truncated NS1 protein [11-13] . However, the isolates do not have a neuraminidase stalk deletion, which is not uncommon in poultry adapted isolates [14].

Deduced amino acid sequences of the PCS of the 20 HA genes revealed three sequences, two of which are consistent with HP AIV, (PETPKRRK/R and PETPKRKRK/R), which is a mutation primarily observed in AIVs which have been circulating in chickens or turkeys. Field and clinical pathogenesis data are also consistent with these isolates being HP [1,2]. The remaining isolates, 35/Chakwal-01 and 34668/Pak-95, had a deduced PCS which is considered LP based on the OIE definition [15].

Although the functional reason is not known an apparent genetic adaptation of AIV to poultry appears to be the truncation of the NS1 protein [11,12,16]. The full length protein is 230 aa, while the NS1 of the H7N3 viruses isolated 1995 to 2003 are 217aa indicating that these viruses have adapted to poultry. In contrast, two viruses from 2004 (100/Karachi-04-H7N3 from April 2004 and one from Mansehra, Chicken/Pakistan/NARC-1282/2004, isolated in December 2004) have a full-length NS1 protein of 230aa. The two viruses with the full length NS1 proteins do not group in the main clade of the NS genes from H7N3 viruses from Pakistan, indicating they came from a separate introduction.

## Conclusions

This report describes the genetic relationships among the H7N3 AIVs in Pakistan and their reassortment with wild bird viruses and the H9N2 AIVs in poultry in Pakistan. Although the specific sources of these viruses can not be known, sporadic isolations since their introduction suggest that despite vigorous control measures in commercial poultry there have been unidentified reservoirs within the country that have an epidemiological link with commercial poultry from different compartments (broilers, broiler breeders, table egg layers). The behavior of H7 viruses in Pakistan varied among the different regions of Pakistan during these outbreaks as some were controlled faster than others, this is likely to be due in at least part to local differences in vaccine use and control programs. The persistence of a single H7 lineage which causes sporadic outbreaks in geographically different regions of Pakistan suggests that there is a reservoir for which transmission is not completely controlled by vaccination and biosecurity, possibly backyard poultry. A reservoir in Pakistan is further supported by the genetic distance between the isolates in Pakistan and other recent isolates from the same region as the viruses in Pakistan have been able to evolve into a "Pakistani" lineage. Because outbreaks with these and other AIVs have substantial economic impact in poultry, virus surveillance and uniform control activities need to be implemented on a sustainable basis in Pakistan. Additionally, recognition of any genetic changes in the circulating AIVs will help to implement better and more targeted control programs.

## Methods

### H7N3 AIV Isolates

Six AIV isolates (Table 1) were selected from the repository of National Reference Laboratory for Poultry Diseases (NRLPD) at the National Agricultural Research Centre (NARC), Islamabad, Pakistan. The isolates were selected to represent different times of isolation, different sectors of poultry production and different geographical origins as described below. An additional 7 isolates from 2004 from the Sindh Poultry Vaccine Centre (SPVC), Karachi, Pakistan were included (Table 1). All isolates were propagated in specific pathogen free embryonating chicken eggs by standard procedures [17].

Isolate 01/Murree-95 was from Murree (Salgran) about 30 Km East of the federal capital, Islamabad, which is densely populated with broiler breeders. Isolates 35/Chakwal-01, 46/Chakwal-03 and 148/Chakwal-04 were from the Chakwal region (the Central Region of Pakistan), which is about 100 Km to the Southwest of the Islamabad. Almost all type of poultry populations (broiler, layer, breeder and backyard) are reared in this region. The isolates 35/Chakwal-01 and 148/Chakwal-04 were from commercial layer flocks while isolate 46/Chakwal-03 was from a broiler flock. The remaining nine isolates were from the Karachi area, which is the Southern Coastal Region of Pakistan, located about 1,050 Km from Chakwal and 1,170 Km from Murree. More than 70% of the country's total commercial layer population is located in this region.

The AIV vaccination status of the flocks from which the isolates in the NRLPD repository originated is known. Three isolates, 01/Murree/95, 23/Karachi-03 and 46/Chakwal/03, were from the poultry flocks not vaccinated against AIV, while 35/Chakwal-01, 148/Chakwal-04 and 100/Karachi-04 were from vaccinated flocks.

### Sequencing

Sequencing and analysis was conducted at Southeast Poultry Research Laboratory, US Department of Agriculture, Agricultural Research Service. Full genome sequence was generated for the 6 isolates from the NRLPD repository, while only the HA and NA genes of the 7 Karachi 2004 isolates from the SPVC repository were sequenced.

RNA was extracted from egg fluids with Trizol LS reagent (Invitrogen, Inc., Carlsbad, CA) in accordance with the manufacturer's instructions. Sequencing templates for individual influenza genes were produced by amplifying the full coding region of each gene by RT-PCR as previously described [18]. Templates were then purified by agarose gel extraction with the QIA-quick gel extraction kit (Qiagen, Inc., Valencia CA). The BigDye terminator kit (Applied Biosystems, Foster City, CA) was used for cycle sequencing and subsequently run on an AB 3730 (Applied Biosystems, Foster City, CA). GenBank accession numbers are given in Table 1.

### Phylogenetic and Sequence Analysis

Phylogenetic analysis included any available sequence data from H7N3 isolates from Pakistan (therefore different numbers of each gene were analyzed), other closely related isolates based nucleotide sequence BLAST search, and numerous sequences from type A influenza isolates from numerous species, dates and regions. The trees shown here were constructed with selected isolates of different lineages to show the genes both in larger context of type A influenza and more closely related isolates. Sequences were aligned with Clustal V (Lasergene, V. 8.0.2 DNAStar, Madison WI). Trees were constructed with merged duplicate runs of BEAST v. 1.4.8 [19] using HKY substitution, empirical base frequency, gamma heterogeneity, codon 2 partitions, relaxed lognormal clock, Yule process tree prior with default operators with unweighted pair group mean with arithmetic average starting tree and a Markov Chain Monte Carlo length of 10^7^.

## Competing interests

The authors declare that they have no competing interests.

## Authors' contributions

MAA, ZA, LS and NS produced and analyzed the sequence data. ES was involved in experimental design, performed the phylogenetic analysis and sequence analysis. DES, KN, AH and SR were involved in experimental design including providing the isolates and associated information. All authors have read an approved the final manuscript.

## Supplementary Material

Additional file 1 **Distance matrix of HA genes shown in figure **1. Similarity (upper triangle) and divergence (lower triangle) of influenza virus H7 HA genes from Paksitani H7N3 isolates and other selected isolates. Click here for file

Additional file 2 **Distance matrix of NA genes shown in figure **2. Similarity (upper triangle) and divergence (lower triangle) of influenza virus N3 NA genes from Paksitani H7N3 isolates and other selected isolates. Click here for file

Additional file 3 **Distance matrix of NS genes shown in figure **3. Similarity (upper triangle) and divergence (lower triangle) of influenza virus NS genes from Paksitani H7N3 isolates and other selected isolates. Click here for file

Additional file 4 **Distance matrix of M genes shown in figure **4. Similarity (upper triangle) and divergence (lower triangle) of influenza virus M genes from Paksitani H7N3 isolates and other selected isolates. Click here for file

Additional file 5 **Distance matrix of NP genes shown in figure **5. Similarity (upper triangle) and divergence (lower triangle) of influenza virus NP genes from Paksitani H7N3 isolates and other selected isolates. Click here for file

Additional file 6 **Distance matrix of PA genes shown in figure **6. Similarity (upper triangle) and divergence (lower triangle) of influenza virus PA genes from Paksitani H7N3 isolates and other selected isolates.
Click here for file

Additional file 7 **Distance matrix of PB1 genes shown in figure **7. Similarity (upper triangle) and divergence (lower triangle) of influenza virus PB1 genes from Paksitani H7N3 isolates and other selected isolates. Click here for file

Additional file 8 **Distance matrix of PB2 genes shown in figure **8. Similarity (upper triangle) and divergence (lower triangle) of influenza virus PB2 genes from Paksitani H7N3 isolates and other selected isolates. Click here for file
