# Supplementary material for: Sequence and phylogenetic analysis of H7N3 avian influenza viruses isolated from poultry in Pakistan 1995-2004
Source: Virol J. 2010 Jun 24;7:137. doi: 10.1186/1743-422X-7-137 (PMC2901269; doi:10.1186/1743-422X-7-137)
Supplement: Additional file 1 — Distance matrix of HA genes shown in figure 1. Similarity (upper triangle) and divergence (lower triangle) of influenza virus H7 HA genes from Paksitani H7N3 isolates and other selected isolates. [file 1743-422X-7-137-S1.PDF]

Additional file 1. Similarity (upper triangle) and divergence (lower triangle) of influenza virus H7 HA genes from Paksitani H7N3 isolates and other selected isolates.

	NARC-01/95	34668/95	34669/95	447/95	CR2/95	16/99	NARC-35/01	NARC-68/02	NARC-72/02	NARC-23/03	NARC-46/04	SPVC-1/04	SPVC-2/04	SPVC-3/04	SPVC-4/04	SPVC-5/04	SPVC-6/04	SPVC-7/04	NARC-100/04	NARC-148/04	Falcon/UAE/98	HK/293/78	Nanchang/1904	Rostock/34	England/63	Italy/1067/99	NL/12/00	N.Ireland/73	Victoria/75	Victoria/85	Victoria/92	Queensland/94	OH/421/87	NY/4450/94	BC/04	176822/02	
Chicken/Murree/NARC-01/1995 H7N3	***	90.2	99.5	99.5	99.9	99.6	90.1	99.9	99.9	99.9	99.9	98.6	98.5	98.5	99.2	99.1	99.1	99.2	99.1	99.1	97.7	88.6	93	82.7	89.1	91.5	91.6	90.2	83.6	82.9	82.8	82.2	77	75.7	75.5	75.7	Chicken/Murree/NARC-01/1995 H7N3
Chicken/Pakistan/34668/1995 H7N3	10.6	***	90.1	90.2	90.3	89.9	99.6	90.1	90	90.1	90.1	89.8	89.6	89.6	90	90	89.9	90	89.8	89.8	88.7	93.2	91.6	83.9	94.1	89.8	89.8	99.8	85.1	84.1	84.1	83.7	77	76.5	76.3	75	Chicken/Pakistan/34668/1995 H7N3
Chicken/Pakistan/34669/1995 H7N3	0.5	10.7	***	99.9	99.6	99.4	89.9	99.5	99.5	99.5	99.5	98.6	98.5	98.4	99.1	99.1	99.1	99.1	98.9	99	97.9	88.6	93	82.6	89.3	91.5	91.5	90.1	83.4	82.7	82.6	81.9	76.9	75.6	75.3	75.5	Chicken/Pakistan/34669/1995 H7N3
Chicken/Pakistan/447/1995 H7N3	0.5	10.6	0.1	***	99.6	99.5	90	99.5	99.4	99.4	99.4	98.5	98.4	98.3	99.1	99	99	99.1	98.9	98.9	98	88.6	93.1	82.6	89.2	91.5	91.6	90.2	83.5	82.8	82.7	81.9	77	75.7	75.3	75.5	Chicken/Pakistan/447/1995 H7N3
Chicken/Pakistan/CR2/1995 H7N3	0.1	10.5	0.4	0.4	***	99.6	90.1	99.8	99.8	99.8	99.8	98.8	98.6	98.6	99.3	99.2	99.2	99.4	99.2	99.2	97.7	88.8	93.1	82.8	89.3	91.7	91.7	90.3	83.7	83	82.9	82.2	77	75.7	75.5	75.6	Chicken/Pakistan/CR2/1995 H7N3
Chicken/Pakistan/16/1999 H7N3	0.4	11.1	0.6	0.5	0.4	***	89.7	99.5	99.4	99.5	99.5	98.5	98.3	98.3	99.2	99.1	99.2	99.2	98.9	99	97.6	88.2	92.1	81	88.6	90.5	90.4	89.9	81.8	81.5	80.8	80.2	74.2	72.2	72.2	72.9	Chicken/Pakistan/16/1999 H7N3
Chicken/Chakwal/NARC-35/2001	10.8	0.2	10.9	10.9	10.7	11.3	***	90	89.8	90	90	89.6	89.5	89.4	89.8	89.8	89.7	89.8	89.6	89.6	88.5	93.1	91.7	83.8	94.2	89.8	89.8	99.7	85.1	84.2	84.2	83.8	76.7	76.3	76	75.1	Chicken/Chakwal/NARC-35/2001
Chicken/Rawalpindi/NARC-68/2002 H7N7	0.1	10.7	0.5	0.5	0.2	0.5	10.8	***	99.9	99.9	99.9	98.7	98.6	98.5	99.2	99.2	99.2	99.2	99	99.1	97.7	88.6	92.9	82.7	89.2	91.5	91.5	90.1	83.5	82.9	82.8	82.1	76.9	75.6	75.4	75.6	Chicken/Rawalpindi/NARC-68/2002 H7N7
Chicken/Rawalpindi/NARC-72/2002 H7N7	0.1	10.8	0.5	0.5	0.2	0.5	11	0	***	99.9	99.9	98.6	98.5	98.5	99.2	99.2	99.1	99.1	98.9	99	97.6	88.5	92.8	82.5	89	91.3	91.4	90	83.4	82.7	82.6	82	76.9	75.6	75.4	75.5	Chicken/Rawalpindi/NARC-72/2002 H7N7
Chicken/Karachi/NARC-23/2003 H7N3	0.1	10.7	0.5	0.6	0.2	0.5	10.9	0.1	0.1	***	100	98.6	98.5	98.5	99.2	99.1	99.1	99.1	98.9	99.1	97.7	88.6	92.9	82.8	89.2	91.5	91.5	90.1	83.5	82.9	82.8	82.1	76.9	75.6	75.4	75.5	Chicken/Karachi/NARC-23/2003 H7N3
Chicken/Chakwal/NARC-46/2003 H7N3	0.1	10.7	0.5	0.6	0.2	0.5	10.9	0.1	0.1	0	***	98.6	98.5	98.5	99.2	99.1	99.1	99.1	98.9	99.1	97.7	88.6	92.9	82.8	89.2	91.5	91.5	90.1	83.5	82.9	82.8	82.1	76.9	75.6	75.4	75.5	Chicken/Chakwal/NARC-46/2003 H7N3
Chicken/Karachi/SPVC-1/2004 H7N3	1.4	11.1	1.4	1.5	1.3	1.5	11.4	1.3	1.3	1.4	1.4	***	99.6	99.6	99	99	98.9	99.1	98.8	98.6	96.7	88.2	92	82.3	88.8	90.6	90.7	89.8	83.2	82.6	82.5	81.9	76.8	75.5	75.2	75.3	Chicken/Karachi/SPVC-1/2004 H7N3
Chicken/Karachi/SPVC-2/2004 H7N3	1.5	11.3	1.6	1.6	1.4	1.7	11.5	1.4	1.4	1.5	1.5	0.4	***	99.9	98.9	98.9	98.8	98.9	98.8	98.5	96.6	88	91.8	82.3	88.7	90.5	90.5	89.6	83.1	82.4	82.3	81.7	76.9	75.6	75.4	75.3	Chicken/Karachi/SPVC-2/2004 H7N3
Chicken/Karachi/SPVC-3/2004 H7N3	1.6	11.4	1.6	1.7	1.4	1.7	11.6	1.5	1.5	1.6	1.6	0.4	0.1	***	98.8	98.8	98.8	98.9	98.7	98.5	96.6	88	91.8	82.2	88.6	90.4	90.5	89.6	83.1	82.3	82.2	81.7	76.8	75.5	75.3	75.3	Chicken/Karachi/SPVC-3/2004 H7N3
Chicken/Karachi/SPVC-4/2004 H7N3	0.8	10.8	0.9	1	0.7	0.9	11.1	0.8	0.7	0.8	0.8	1	1.1	1.2	***	99.9	99.4	99.5	99.4	99.2	97.3	88.5	92.5	82.5	89	91.1	91.1	89.9	83.2	82.6	82.5	82	76.9	75.6	75.4	75.5	Chicken/Karachi/SPVC-4/2004 H7N3
Chicken/Karachi/SPVC-5/2004 H7N3	0.8	10.7	0.8	0.9	0.7	0.8	11	0.7	0.7	0.8	0.8	0.9	1	1.1	0	***	99.3	99.4	99.4	99.1	97.2	88.5	92.4	82.5	89	91	91.1	89.9	83.2	82.6	82.5	82	76.9	75.6	75.4	75.4	Chicken/Karachi/SPVC-5/2004 H7N3
Chicken/Karachi/SPVC-6/2004 H7N3	0.9	11	1	1	0.8	0.9	11.2	0.8	0.8	0.9	0.9	1.1	1.2	1.3	0.7	0.6	***	99.8	99.1	99.5	97.3	88.3	92.5	82.4	88.9	91.1	91.1	89.9	83.2	82.6	82.5	82	76.6	75.3	75.1	75.2	Chicken/Karachi/SPVC-6/2004 H7N3
Chicken/Karachi/SPVC-7/2004 H7N3	0.8	10.8	1	0.9	0.7	0.8	11.1	0.8	0.8	0.9	0.9	1	1.1	1.1	0.5	0.5	0.2	***	99.4	99.5	97.4	88.5	92.6	82.4	88.9	91.2	91.2	90	83.3	82.7	82.6	82.1	76.7	75.4	75.2	75.3	Chicken/Karachi/SPVC-7/2004 H7N3
Chicken/Karachi/NARC-100/2004 H7N3	0.9	11.1	1.1	1.1	0.8	1.1	11.3	1	1	1.1	1.1	1.3	1.3	1.3	0.6	0.5	0.9	0.7	***	98.9	97	88.3	92.4	82.3	88.7	90.9	91.1	89.8	83.2	82.5	82.3	81.7	76.7	75.3	75.1	75.3	Chicken/Karachi/NARC-100/2004 H7N3
Chicken/Chakwal/NARC-148/2004 H7N3	0.9	11.1	1	1.1	0.8	1	11.3	0.9	0.9	0.9	0.9	1.4	1.5	1.6	0.8	0.8	0.5	0.5	1.1	***	97.1	88.4	92.4	82.4	89	91	91.1	89.8	83.2	82.6	82.6	82	76.7	75.6	75.3	75.2	Chicken/Chakwal/NARC-148/2004 H7N3
Falcon/UAE/188-2384/1998 H7N3	2.3	12.5	2.1	2	2.3	2.5	12.8	2.4	2.4	2.4	2.4	3.4	3.5	3.5	2.8	2.7	2.8	2.7	3	2.9	***	87	91.2	81.3	87.9	90	90	88.7	81.8	81.5	80.8	80.4	74.8	72.6	72.3	73.5	Falcon/UAE/188-2384/1998 H7N3
Duck/HongKong/293/1978 H7N2	12.7	7.2	12.8	12.7	12.5	13.2	7.3	12.8	12.8	12.8	12.8	13.3	13.4	13.5	12.9	12.8	13.1	12.9	13	13	14.8	***	89.7	84.3	93.3	88.6	88.6	93.3	84.4	84.1	83.6	83.4	76.2	75.7	75.4	74.7	Duck/HongKong/293/1978 H7N2
Duck/Nanchang/1904/1992 H7N2	7.5	9	7.5	7.4	7.4	8.5	8.9	7.6	7.7	7.6	7.6	8.7	8.8	8.9	8.1	8	8.1	7.9	8.2	8.1	9.5	11.4	***	83.9	90.4	95.6	95.4	91.8	84.1	83.3	83.2	82.7	77	76.5	76.7	75.8	Duck/Nanchang/1904/1992 H7N2
Chicken/Rostock/1934 H7N1	20.5	18.8	20.6	20.6	20.3	22.8	18.9	20.4	20.7	20.3	20.3	21	21.1	21.2	20.7	20.6	20.8	20.8	21	20.9	22.4	18.3	18.8	***	86.4	83.7	83.6	84	84.4	83.6	83	82.7	76.2	75.7	75.9	75.8	Chicken/Rostock/1934 H7N1
Turkey/Egngland/1963 H7N3	12	6.1	11.9	11.9	11.9	12.6	6	11.9	12.1	11.9	11.9	12.5	12.6	12.7	12.2	12.1	12.3	12.3	12.5	12.2	13.5	7.2	10.5	15.5	***	88.6	88.8	94.3	86.1	84.9	84.6	84.7	76.6	76	75.6	75.3	Turkey/Egngland/1963 H7N3
Chicken/Italy/1067/1999 H7N1	9.2	11.1	9.2	9.2	9	10.3	11.1	9.2	9.3	9.2	9.2	10.3	10.4	10.5	9.7	9.7	9.7	9.6	9.9	9.8	11	12.8	4.6	19	12.8	***	98.6	90.1	84.1	83.4	83.3	82.7	77.2	76.7	76.7	76.1	Chicken/Italy/1067/1999 H7N1
Mallard/Netherlands/12/00 H7N3	9.1	11.2	9.2	9.1	9	10.5	11.2	9.2	9.3	9.2	9.2	10.2	10.4	10.4	9.6	9.6	9.6	9.5	9.7	9.7	11	12.7	4.8	19.2	12.6	1.4	***	90	84.1	83.5	83.3	82.7	77.3	76.6	76.7	75.7	Mallard/Netherlands/12/00 H7N3
Parrot/NorthIreland/VF-73-67/1973 H7N1	10.8	0.1	10.8	10.8	10.6	11.1	0.2	10.8	11	10.8	10.8	11.3	11.4	11.5	11	10.9	11.1	11	11.2	11.2	12.6	7.1	8.8	18.8	6	11	11	***	85.1	84.2	84.1	83.7	77	76.4	76.2	75	Parrot/NorthIreland/VF-73-67/1973 H7N1
Chicken/Victoria/1975 H7NN7	19.1	17	19.4	19.4	19	21.7	17	19.2	19.3	19.2	19.2	19.6	19.8	19.9	19.6	19.5	19.7	19.5	19.7	19.6	21.7	18.1	18.5	18.1	15.9	18.5	18.5	17.1	***	95.3	92.7	91.5	76.4	75.9	75.9	76.2	Chicken/Victoria/1975 H7NN7
Chicken/Victoria/1/1985 H7N7	20.1	18.3	20.4	20.3	20	22.2	18.2	20.2	20.3	20.2	20.2	20.6	20.8	20.9	20.6	20.4	20.6	20.4	20.8	20.5	22	18.6	19.6	19.3	17.5	19.5	19.4	18.4	4.9	***	95.4	94.4	75.7	74.9	74.7	75.4	Chicken/Victoria/1/1985 H7N7
Chicken/Victoria/224/1992 H7N3	20.2	18.4	20.6	20.5	20.1	23.1	18.3	20.3	20.5	20.3	20.3	20.7	21	21	20.6	20.5	20.7	20.5	21	20.5	23.1	19.2	19.8	20	17.8	19.6	19.6	18.5	7.8	4.8	***	96.5	75.1	74.5	74.5	75.7	Chicken/Victoria/224/1992 H7N3
Chicken/Queensland/1984 H7N3	21.2	19	21.6	21.5	21.1	24	18.9	21.3	21.4	21.3	21.3	21.5	21.8	21.9	21.4	21.3	21.4	21.3	21.8	21.3	23.7	19.6	20.5	20.5	17.8	20.6	20.6	19.1	9.2	5.9	3.6	***	74.8	74.1	74	75.6	Chicken/Queensland/1984 H7N3
Mallard/OH/421/1987 H7N8	27.8	27.7	27.9	27.8	27.8	32.1	28	27.9	27.8	28	28	28.1	28	28.1	27.9	27.8	28.4	28.2	28.3	28.2	31.2	29	27.8	29	28.4	27.5	27.3	27.8	28.9	29.8	30.9	31.4	***	93.5	92	82.4	Mallard/OH/421/1987 H7N8
Turkey/NY/4450/1994 H7N2	29.9	28.5	30	29.9	29.9	35.5	28.7	29.9	29.9	29.9	29.9	30.1	30	30.1	29.9	29.9	30.4	30.2	30.4	29.9	34.8	29.9	28.6	29.7	29.3	28.3	28.5	28.7	29.7	31.2	31.9	32.5	6.9	***	95.2	81.3	Turkey/NY/4450/1994 H7N2
Chicken/BritishColumbia/2004 H7N3	30.2	28.8	30.5	30.4	30.2	35.5	29.2	30.3	30.2	30.3	30.3	30.7	30.4	30.5	30.3	30.2	30.8	30.6	30.7	30.5	35.4	30.3	28.3	29.3	29.9	28.3	28.3	29	29.5	31.6	31.8	32.7	8.7	5	***	81.3	Chicken/BritishColumbia/2004 H7N3
Chicken/chile/176822/2002 H7N3	29.8	30.8	30.1	30	29.9	34.3	30.6	29.9	30	30	30	30.5	30.4	30.5	30.1	30.1	30.6	30.4	30.4	30.6	33.3	31.4	29.6	29.4	30.4	29.2	29.9	31	28.9	30.2	29.7	30	20.8	22.2	22.3	***	Chicken/chile/176822/2002 H7N3
	NARC-01/95	34668/95	34669/95	447/95	CR2/95	16/99	NARC-35/01	NARC-68/02	NARC-72/02	NARC-23/03	NARC-46/04	SPVC-1/04	SPVC-2/04	SPVC-3/04	SPVC-4/04	SPVC-5/04	SPVC-6/04	SPVC-7/04	NARC-100/04	NARC-148/04	Falcon/UAE/98	HK/293/78	Nanchang/1904	Rostock/34	England/63	Italy/1067/99	NL/12/00	N.Ireland/73	Victoria/75	Victoria/85	Victoria/92	Queensland/94	OH/421/87	NY/4450/94	BC/04	176822/02	
