# Supplementary material for: Sequence and phylogenetic analysis of H7N3 avian influenza viruses isolated from poultry in Pakistan 1995-2004
Source: Virol J. 2010 Jun 24;7:137. doi: 10.1186/1743-422X-7-137 (PMC2901269; doi:10.1186/1743-422X-7-137)
Supplement: Additional file 2 — Distance matrix of NA genes shown in figure 2. Similarity (upper triangle) and divergence (lower triangle) of influenza virus N3 NA genes from Paksitani H7N3 isolates and other selected isolates. [file 1743-422X-7-137-S2.PDF]

Additional file 2. Similarity (upper triangle) and divergence (lower triangle) of influenza virus N3 NA genes from Paksitani H7N3 isolates and other selected isolates.

	NARC-01/95	Pak/34668/95	Pak/34669/95	Pak/447/95	NARC-35/01	NARC-68/02	NARC-72/02	NARC-23/03	NARC-46/04	NARC-148/04	SPVC-1/04	SPVC-2/04	SPVC-3/04	SPVC-4/04	SPVC-5/04	SPVC-6/04	SPVC-7/04	NARC-100/04	Tern/61	England/63	HK/205/77	Victoria/92	Queensland/94	NL/12/00	BC/04	176822/02	
Chicken/Murree/NARC-01/1995 H7N3	***	99.6	99.7	99.8	100	99.5	99.5	99.9	99.9	99.9	99.7	99.6	99.6	99.6	99.6	99.9	99.9	99.4	90.9	84.5	92.2	90.3	90.4	94.2	85.7	83.1	Chicken/Murree/NARC-01/1995 H7N3
Chicken/Pakistan/34668/1995 H7N3	0.3	***	99.3	99.3	99.6	99.5	99.5	99.5	99.5	99.5	99.3	99.2	99.2	99.2	99.2	99.5	99.5	98.9	90.8	84.2	92.1	90	90.2	93.9	85.6	83.1	Chicken/Pakistan/34668/1995 H7N3
Chicken/Pakistan/34669/1995 H7N3	0.3	0.6	***	100	99.7	99.2	99.2	99.6	99.6	99.6	99.4	99.4	99.4	99.3	99.3	99.6	99.6	99.1	91.1	84.4	92.2	90.4	90.6	94.3	85.8	83.3	Chicken/Pakistan/34669/1995 H7N3
Chicken/Pakistan/447/1995 H7N3	0.2	0.4	0	***	99.8	99.3	99.3	99.7	99.7	99.7	99.4	99.3	99.3	99.3	99.3	99.8	99.8	99	90.4	80.5	92.1	89.6	89.9	94	85.9	83.1	Chicken/Pakistan/447/1995 H7N3
Chicken/Chakwal/NARC-35/2001	0	0.3	0.3	0.2	***	99.5	99.5	99.9	99.9	99.9	99.7	99.6	99.6	99.6	99.6	99.9	99.9	99.4	90.9	84.5	92.2	90.3	90.4	94.2	85.7	83.1	Chicken/Chakwal/NARC-35/2001
Chicken/Rawalpindi/NARC-68/2002 H7N7	0.4	0.2	0.6	0.6	0.4	***	100	99.5	99.5	99.5	99.2	99.2	99.2	99.1	99.1	99.4	99.4	98.9	90.9	84.3	92.3	90.1	90.2	93.8	85.8	83.1	Chicken/Rawalpindi/NARC-68/2002 H7N7
Chicken/Rawalpindi/NARC-72/2002 H7N7	0.4	0.2	0.6	0.6	0.4	0	***	99.5	99.5	99.5	99.2	99.2	99.2	99.1	99.1	99.4	99.4	98.9	90.9	84.3	92.3	90.1	90.2	93.8	85.8	83.1	Chicken/Rawalpindi/NARC-72/2002 H7N7
Chicken/Karachi/NARC-23/2003 H7N3	0.1	0.4	0.4	0.3	0.1	0.4	0.4	***	100	100	99.6	99.6	99.6	99.5	99.5	99.9	99.9	99.3	90.9	84.4	92.1	90.2	90.4	94.1	85.7	83.1	Chicken/Karachi/NARC-23/2003 H7N3
Chicken/Chakwal/NARC-46/2003 H7N3	0.1	0.4	0.4	0.3	0.1	0.4	0.4	0	***	100	99.6	99.6	99.6	99.5	99.5	99.9	99.9	99.3	90.9	84.4	92.1	90.2	90.4	94.1	85.7	83.1	Chicken/Chakwal/NARC-46/2003 H7N3
Chicken/Chakwal/NARC-148/2004 H7N3	0.1	0.4	0.4	0.3	0.1	0.4	0.4	0	0	***	99.6	99.6	99.6	99.5	99.5	99.9	99.9	99.3	90.9	84.4	92.1	90.2	90.4	94.1	85.7	83.1	Chicken/Chakwal/NARC-148/2004 H7N3
Chicken/Karachi/SPVC-1/2004 H7N3	0.3	0.6	0.6	0.6	0.3	0.6	0.6	0.4	0.4	0.4	***	99.8	99.8	99.4	99.4	99.8	99.8	99.4	90.7	84.3	92.1	90	90.2	94	85.4	82.9	Chicken/Karachi/SPVC-1/2004 H7N3
Chicken/Karachi/SPVC-2/2004 H7N3	0.4	0.6	0.6	0.7	0.4	0.7	0.7	0.4	0.4	0.4	0.2	***	100	99.4	99.4	99.7	99.7	99.3	90.7	84.3	92.1	90	90.1	94	85.3	82.9	Chicken/Karachi/SPVC-2/2004 H7N3
Chicken/Karachi/SPVC-3/2004 H7N3	0.4	0.6	0.6	0.7	0.4	0.7	0.7	0.4	0.4	0.4	0.2	0	***	99.4	99.4	99.7	99.7	99.3	90.7	84.3	92.1	90	90.1	94	85.3	82.9	Chicken/Karachi/SPVC-3/2004 H7N3
Chicken/Karachi/SPVC-4/2004 H7N3	0.4	0.7	0.7	0.7	0.4	0.8	0.8	0.5	0.5	0.5	0.6	0.6	0.6	***	100	99.6	99.6	99.5	90.7	84.2	92.1	90	90.2	94.1	85.4	83	Chicken/Karachi/SPVC-4/2004 H7N3
Chicken/Karachi/SPVC-5/2004 H7N3	0.4	0.7	0.7	0.7	0.4	0.8	0.8	0.5	0.5	0.5	0.6	0.6	0.6	0	***	99.6	99.6	99.5	90.7	84.2	92.1	90	90.2	94.1	85.4	83	Chicken/Karachi/SPVC-5/2004 H7N3
Chicken/Karachi/SPVC-6/2004 H7N3	0.1	0.4	0.4	0.2	0.1	0.4	0.4	0.1	0.1	0.1	0.2	0.3	0.3	0.4	0.4	***	100	99.4	90.9	84.4	92.3	90.2	90.4	94.1	85.6	83.1	Chicken/Karachi/SPVC-6/2004 H7N3
Chicken/Karachi/SPVC-7/2004 H7N3	0.1	0.4	0.4	0.2	0.1	0.4	0.4	0.1	0.1	0.1	0.2	0.3	0.3	0.4	0.4	0	***	99.4	90.9	84.4	92.3	90.2	90.4	94.1	85.6	83.1	Chicken/Karachi/SPVC-7/2004 H7N3
Chicken/Karachi/NARC-100/2004 H7N3	0.6	0.9	0.9	1	0.6	1	1	0.7	0.7	0.7	0.6	0.7	0.7	0.5	0.5	0.6	0.6	***	90.4	84.1	91.9	89.8	90	93.9	85.2	82.7	Chicken/Karachi/NARC-100/2004 H7N3
Tern/SouthAfrica/61 H5N3	10	10	9.8	10.6	10	9.9	9.9	10	10	10	10.3	10.2	10.2	10.3	10.3	10	10	10.5	***	89	95	92.6	92.2	92.1	87.5	84.4	Tern/SouthAfrica/61 H5N3
Turkey/Egngland/1963 H7N3	11.4	11.6	11.5	12.6	11.4	11.5	11.5	11.5	11.5	11.5	11.6	11.5	11.5	11.7	11.7	11.5	11.5	11.9	5.8	***	86.9	84.8	85.5	85	82.3	79.8	Turkey/Egngland/1963 H7N3
Duck/HongKong/205/77 H5N3	8.5	8.5	8.5	8.5	8.5	8.2	8.2	8.6	8.6	8.6	8.6	8.6	8.6	8.6	8.6	8.4	8.4	8.9	5.3	8.3	***	92.6	92.3	93.3	87.1	84.1	Duck/HongKong/205/77 H5N3
Chicken/Victoria/224/1992 H7N3	10.7	10.9	10.5	11.5	10.7	10.8	10.8	10.8	10.8	10.8	11	11.1	11.1	11.1	11.1	10.8	10.8	11.3	8	10.9	8.1	***	97.6	91.5	85.5	83.1	Chicken/Victoria/224/1992 H7N3
Chicken/Queensland/1994 H7N3	10.5	10.7	10.4	11.1	10.5	10.6	10.6	10.6	10.6	10.6	10.9	11	11	10.9	10.9	10.6	10.6	11.1	8.5	10	8.4	2.5	***	91.8	86	83.1	Chicken/Queensland/1994 H7N3
Mallard/Netherlands/12/00 H7N3	6.1	6.3	6	6.3	6.1	6.4	6.4	6.2	6.2	6.2	6.3	6.4	6.4	6.2	6.2	6.2	6.2	6.4	8.5	10.7	7.2	9.2	8.9	***	86	83.9	Mallard/Netherlands/12/00 H7N3
Chicken/BritishColumbia/2004 H7N3	16.5	16.5	16.3	16.1	16.5	16.2	16.2	16.4	16.4	16.4	16.9	17	17	16.9	16.9	16.6	16.6	17.2	14.2	14.4	14.8	16.8	16.1	16.1	***	84.6	Chicken/BritishColumbia/2004 H7N3
Chicken/chile/176822/02 H7N3	19.9	19.8	19.7	19.9	19.9	19.9	19.9	19.9	19.9	19.9	20.3	20.2	20.2	20.1	20.1	20	20	20.5	18.3	17.9	18.6	20	19.9	18.8	18.1	***	Chicken/chile/176822/02 H7N3
	NARC-01/95	Pak/34668/95	Pak/34669/95	Pak/447/95	NARC-35/01	NARC-68/02	NARC-72/02	NARC-23/03	NARC-46/04	NARC-148/04	SPVC-1/04	SPVC-2/04	SPVC-3/04	SPVC-4/04	SPVC-5/04	SPVC-6/04	SPVC-7/04	NARC-100/04	Tern/61	England/63	HK/205/77	Victoria/92	Queensland/94	NL/12/00	BC/04	176822/02	
