# Supplementary material for: Sequence and phylogenetic analysis of H7N3 avian influenza viruses isolated from poultry in Pakistan 1995-2004
Source: Virol J. 2010 Jun 24;7:137. doi: 10.1186/1743-422X-7-137 (PMC2901269; doi:10.1186/1743-422X-7-137)
Supplement: Additional file 8 — Distance matrix of PB2 genes shown in figure 8. Similarity (upper triangle) and divergence (lower triangle) of influenza virus PB2 genes from Paksitani H7N3 isolates and other selected isolates. [file 1743-422X-7-137-S8.PDF]

Additional file 8. Similarity (upper triangle) and divergence (lower triangle) of influenza virus PB2 genes from Paksitani H7N3 isolates and other selected isolates.

	NARC-01/95	Pak/34668/95	Pak/34669/95	Pak/447/95	Pak/2/99	NARC-35/01	NARC-68/02	NARC-72/02	NARC-23/03	NARC-46/04	NARC-100/04	NARC-148/04	UDL-02/06	Dubai/303/00	HK/205/77	HK/293/78	HK/702/79	Nanchang/1749	Nanchang/1904	Guandong/96	HK/G9/97	HK/483/97	Victoria/92	Queensland/94	Rostock/34	England/63	Potsdam/84	England/91	Italy/1067/99	NL/12/00	OH/421/87	NY/4450/94	BC/04	176822/02	
Chicken/Murree/NARC-01/1995 H7N3	***	100	99.7	83.4	87.6	99.9	99.7	99.3	99.8	99.8	99.1	99.8	91.4	92	86.8	86.9	87.1	87.3	86.5	87.3	88.1	87.8	86.6	86	85.4	87.8	94.6	90.1	89.1	89.4	83.2	83	82.6	80.8	Chicken/Murree/NARC-01/1995 H7N3
Chicken/Pakistan/34668/1995 H7N3	0	***	99.7	83.4	87.5	99.9	99.7	99.3	99.8	99.8	99.1	99.8	91.4	92	86.7	86.8	87	87.3	86.4	87.4	88.1	87.8	86.5	86	85.4	87.7	94.6	90	89.1	89.3	83.1	82.9	82.5	80.8	Chicken/Pakistan/34668/1995 H7N3
Chicken/Pakistan/34669/1995 H7N3	0.3	0.3	***	83.2	87.8	99.6	99.7	99.4	99.8	99.8	99.1	99.7	91.4	92.1	87	87	87.3	87.4	86.5	87.5	88.3	88	86.7	86.2	85.6	87.9	94.8	90	89.2	89.4	83.2	83.1	82.5	80.8	Chicken/Pakistan/34669/1995 H7N3
Chicken/Pakistan/447/1995 H7N3	18.1	18.1	18.2	***	82.7	83.4	83.3	82.6	83.5	83.5	82.7	83.5	83.6	84.3	84.7	84.3	84.7	84.9	84.2	84.7	82.3	82.9	84.6	84.6	83.9	84.8	84	84.3	84.5	84.6	90.2	89.5	90	81.4	Chicken/Pakistan/447/1995 H7N3
Chicken/Pakistan/2/1999 H9N2	13.3	13.3	13	19.2	***	87.5	87.7	87.4	87.7	87.7	87.1	87.7	86.8	87.8	86	85.8	86	86.3	85.8	86.8	97.1	97.8	85.7	85.2	85.4	86.8	89.6	89.5	88.4	88.9	82.6	82.6	81.9	79.3	Chicken/Pakistan/2/1999 H9N2
Chicken/Chakwal/NARC-35/2001 H7N3	0.1	0.1	0.4	18	13.4	***	99.6	99.3	99.7	99.7	99	99.7	91.4	91.9	86.7	86.8	87	87.3	86.6	87.3	87.9	87.7	86.6	86	85.4	87.7	94.5	90	89	89.3	83.2	83.1	82.5	80.8	Chicken/Chakwal/NARC-35/2001 H7N3
Chicken/Rawalpindi/NARC-68/2002 H7N7	0.2	0.2	0.2	17.9	13	0.3	***	99.5	99.9	99.9	99.1	99.8	91.4	92.1	86.8	86.9	87.1	87.3	86.4	87.3	88.2	87.9	86.6	86	85.5	87.8	94.7	90	89.2	89.4	83.2	82.9	82.5	80.8	Chicken/Rawalpindi/NARC-68/2002 H7N7
Chicken/Rawalpindi/NARC-72/2002 H7N7	0.2	0.2	0.2	18	13	0.3	0	***	99.5	99.5	98.7	99.5	90.9	91.7	86.4	86.5	86.7	87	86.1	86.9	87.9	87.6	86.2	85.6	85.2	87.4	94.3	89.6	88.7	89	82.8	82.5	82.2	80.3	Chicken/Rawalpindi/NARC-72/2002 H7N7
Chicken/Karachi/NARC-23/2003 H7N3	0.2	0.1	0.2	18	13.1	0.3	0	0	***	100	99.2	100	91.4	92.1	86.9	87	87.2	87.4	86.6	87.4	88.2	87.8	86.7	86.1	85.5	87.9	94.7	90	89.1	89.4	83.3	83	82.6	80.7	Chicken/Karachi/NARC-23/2003 H7N3
Chicken/Chakwal/NARC-46/2003 H7N3	0.2	0.1	0.2	18	13.1	0.3	0	0	0	***	99.2	100	91.4	92.1	86.9	87	87.2	87.4	86.6	87.4	88.2	87.8	86.7	86.1	85.5	87.9	94.7	90	89.1	89.4	83.3	83	82.6	80.7	Chicken/Chakwal/NARC-46/2003 H7N3
Chicken/Karachi/NARC-100/2004 H7N3	0.9	0.8	0.9	18.7	13.7	1	0.8	0.8	0.8	0.8	***	99.1	91.1	91.5	86.4	86.4	86.6	86.8	86	86.9	87.6	87.3	86.2	85.6	85.2	87.3	94	89.7	89	89.2	82.6	82.6	82.2	80.6	Chicken/Karachi/NARC-100/2004 H7N3
Chicken/Chakwal/NARC-148/2004 H7N3	0.2	0.2	0.3	18	13.2	0.3	0.1	0.1	0	0	0.9	***	91.3	92	86.9	86.9	87.2	87.4	86.6	87.4	88.1	87.8	86.6	86.1	85.5	87.8	94.6	90	89.1	89.3	83.2	83	82.6	80.6	Chicken/Chakwal/NARC-148/2004 H7N3
Chicken/Pakistan/UDL-02/2006 H9N2	8.9	8.9	8.9	17.8	14.2	8.9	8.9	9	9	9	9.3	9	***	95.9	87.1	87.3	87.1	87.2	86.8	87.3	87	86.9	86.9	86.8	86.2	88.3	94	90.4	89.4	89.7	82.9	83.9	83.3	80.9	Chicken/Pakistan/UDL-02/2006 H9N2
Quail/Dubai/303/2000 H9N2	8.3	8.3	8.1	17.1	13	8.4	8.1	8.2	8.2	8.2	8.8	8.3	4.2	***	87.2	87.3	87	87.5	86.6	87.6	88	87.8	87	86.5	86.7	88.2	95.2	90.7	90	90.2	83.4	83.7	83.4	80.8	Quail/Dubai/303/2000 H9N2
Duck/HongKong/205/1977 H5N3	14.1	14.1	13.9	16.8	15.3	14.2	13.9	14	14	14	14.4	14	13.5	13.6	***	96.5	96.1	96.2	95.6	94.7	86.4	86.2	95.8	94.6	88	94.9	88.3	88	88.4	89	84.5	84.3	84.1	81.5	Duck/HongKong/205/1977 H5N3
Duck/HongKong/293/1978 H7N2	14.2	14.2	13.9	17.1	15.4	14.2	14	14.1	14.1	14.1	14.5	14.1	13.5	13.7	3.6	***	96.4	96	95.6	94.5	86.1	85.8	95.5	94.5	88.5	95	88.1	87.5	87.9	88.3	84.9	85.3	84.6	81.6	Duck/HongKong/293/1978 H7N2
Duck/HongKong/702/1979 H9N2	13.9	13.9	13.6	16.7	15.2	13.9	13.7	13.8	13.8	13.8	14.2	13.8	13.6	13.9	4	3.7	***	96.2	96.1	94.6	86.4	86.7	95.5	94.1	88.3	94.9	88.1	88.2	88	88.4	84.7	84.2	84	80.9	Duck/HongKong/702/1979 H9N2
Duck/Nanchang/1749/1992 H11N2	13.7	13.8	13.6	16.4	15.1	13.8	13.6	13.6	13.6	13.6	14.2	13.7	13.8	13.5	3.8	4	3.9	***	97.7	95.3	86.3	86.4	96.1	94.9	88.7	94.9	88.4	88.4	88.4	88.6	85	84.8	84.6	81.2	Duck/Nanchang/1749/1992 H11N2
Duck/Nanchang/1904/1992 H7N2	14.7	14.7	14.7	17.3	15.7	14.6	14.6	14.6	14.6	14.6	15.1	14.6	14.2	14.6	4.6	4.5	4	2.3	***	95.1	85.7	85.8	95.8	94.2	87.8	94.1	87.6	87.3	87.4	87.7	84.7	84.3	84.3	80.9	Duck/Nanchang/1904/1992 H7N2
Goose/Guandong/1996 H5N1	13.6	13.6	13.4	16.8	14.1	13.7	13.6	13.6	13.5	13.5	14	13.6	13.5	13.4	5.4	5.6	5.5	4.8	5.1	***	87.1	87.2	94.5	93	87.9	93.2	88.4	87.9	88.4	88.5	84.6	83.6	83.5	80.3	Goose/Guandong/1996 H5N1
Chicken/HongKong/G9/1997 H9N2	12.7	12.6	12.4	19.3	3	12.8	12.4	12.4	12.6	12.6	13	12.6	13.9	12.9	14.8	15.1	14.7	15.1	15.7	13.9	***	97.6	85.6	85.4	86.1	87.1	89.9	89.5	88.4	88.9	82.4	82.5	81.8	79.7	Chicken/HongKong/G9/1997 H9N2
HongKong/483/1997 H5N1	12.9	12.8	12.6	18.9	2.3	13	12.7	12.6	12.8	12.8	13.3	12.9	14	12.9	15.1	15.3	14.4	14.9	15.6	13.8	2.4	***	85.7	85.5	85.8	87.2	89.6	89.7	88.5	88.9	82.9	82.6	82.3	79.6	HongKong/483/1997 H5N1
Chicken/Victoria/224/1992 H7N3	14.4	14.4	14.1	16.8	15.5	14.3	14.3	14.3	14.3	14.3	14.7	14.3	13.8	13.9	4.3	4.6	4.6	3.9	4.4	5.6	15.6	15.5	***	95.9	87.8	94.6	88	88.1	88	88.4	84.5	84.6	84.2	81.4	Chicken/Victoria/224/1992 H7N3
Chicken/Queensland/1994 H7N3	15	15	14.7	16.5	16	15	14.9	14.9	14.9	14.9	15.4	14.9	14.1	14.6	5.6	5.8	6.2	5.3	6	7.4	15.8	15.6	4.2	***	87.6	93.4	87.8	87.8	87.6	88	84.5	84.3	85	81.4	Chicken/Queensland/1994 H7N3
Chicken/Rostock/1934 H7N1	15.9	15.8	15.7	15	15.6	15.9	15.7	15.6	15.8	15.8	16	15.8	14.9	14.2	12.8	12.3	12.5	12.1	13	12.9	14.9	15.2	12.8	13.5	***	89.6	87.2	87	87.2	87.6	84.1	84.5	85	80.4	Chicken/Rostock/1934 H7N1
Turkey/England/1963 H7N3	13	13	12.8	16.5	14.3	13	12.8	12.9	12.9	12.9	13.5	12.9	12.4	12.6	5	5	5.3	5.3	6.1	6.8	13.8	13.8	5.5	6.8	10.9	***	89	89.3	88.9	89.4	85	84.8	85.2	81.6	Turkey/England/1963 H7N3
Duck/Potsdam/2216-4/1984 H5N6	5.5	5.5	5.3	17.2	11	5.6	5.4	5.4	5.4	5.4	6.2	5.5	6.2	4.9	12.4	12.7	12.7	12.5	13.3	12.5	10.8	11	12.7	13	13.7	11.5	***	92.8	92.1	92.2	83.5	83.9	83.6	81	Duck/Potsdam/2216-4/1984 H5N6
Turkey/England/50-92/1991 H5N1	10.6	10.7	10.6	17.4	11.3	10.7	10.6	10.6	10.6	10.6	10.9	10.7	10.2	10	12.9	13.4	12.8	12.7	13.8	13.1	11.1	10.9	12.6	13.2	14	11.4	7.6	***	95	95	84.1	84.6	84.9	80.4	Turkey/England/50-92/1991 H5N1
Chicken/Italy/1067/1999 H7N1	11.5	11.5	11.4	17	12.3	11.7	11.4	11.4	11.6	11.6	11.6	11.6	11.3	10.7	12.2	12.8	12.8	12.4	13.6	12.5	12.3	12.3	12.5	13.3	13.8	11.6	8.3	5.1	***	98.5	83.6	84	84.5	80.3	Chicken/Italy/1067/1999 H7N1
Mallard/Netherlands/12/2000 H7N3	11.4	11.4	11.3	16.8	11.8	11.5	11.2	11.3	11.4	11.4	11.4	11.4	11	10.4	11.7	12.6	12.4	12.2	13.3	12.4	11.8	11.8	12.3	13	13.4	11.2	8.3	5.1	1.6	***	84.2	84.6	85	81	Mallard/Netherlands/12/2000 H7N3
Mallard/OH/421/1987 H7N8	18.4	18.5	18.3	10.5	19.1	18.5	18.2	18.3	18.3	18.3	18.9	18.3	18.8	18.2	17.1	16.5	16.8	16.4	16.8	16.9	19.3	18.7	17	17.1	17.3	16.2	17.9	17.5	18.1	17.5	***	90.7	93.1	82.1	Mallard/OH/421/1987 H7N8
Turkey/NY/4450/1994 H7N2	18.5	18.5	18.3	11.4	18.5	18.4	18.4	18.5	18.5	18.5	18.9	18.5	17.1	17.4	17	15.9	17.2	16.6	17.2	17.8	18.8	18.5	16.8	17.2	16.5	16.4	17.2	16.8	17.5	17	9.8	***	90.6	82.4	Turkey/NY/4450/1994 H7N2
Chicken/BritishColumbia/2004 H7N3	19.1	19.2	19.2	10.6	19.7	19.2	19.1	19.1	19.1	19.1	19.5	19.2	18.2	18	17.4	16.8	17.3	16.9	17.1	17.9	20	19.5	17.2	16.4	16.4	16	17.9	16.7	17	16.6	7.2	10	***	82.5	Chicken/BritishColumbia/2004 H7N3
Chicken/Chile/176822/2002 H7N3	21	21	21.1	20.5	22.7	21	21	21.2	21.2	21.2	21.3	21.3	20.7	20.8	20.5	20.3	20.9	20.8	21	21.9	22.3	22.3	20.4	20.4	21.4	20.3	20.8	21.8	21.7	21	19.8	19.1	19.1	***	Chicken/Chile/176822/2002 H7N3
	NARC-01/95	Pak/34668/95	Pak/34669/95	Pak/447/95	Pak/2/99	NARC-35/01	NARC-68/02	NARC-72/02	NARC-23/03	NARC-46/04	NARC-100/04	NARC-148/04	UDL-02/06	Dubai/303/00	HK/205/77	HK/293/78	HK/702/79	Nanchang/1749	Nanchang/1904	Guandong/96	HK/G9/97	HK/483/97	Victoria/92	Queensland/94	Rostock/34	England/63	Potsdam/84	England/91	Italy/1067/99	NL/12/00	OH/421/87	NY/4450/94	BC/04	176822/02	
